# Long-Term Surgical Outcomes and Pathological Analysis of Proctectomy Specimens after Subtotal Colectomy for Ulcerative Colitis: A Retrospective Cohort Study from a Tertiary Centre

**DOI:** 10.3390/jcm12175729

**Published:** 2023-09-02

**Authors:** Kethaki Prathivadi Bhayankaram, Jeremy Meyer, Boby Sebastian, Justin Davies, James Wheeler

**Affiliations:** 1 Cambridge Colorectal Unit, Addenbrooke’s Hospital, Cambridge University Hospitals NHS Foundation Trust, Cambridge CB2 0QQ, UK; kethaki.pb@doctors.org.uk (K.P.B.);; 2 West Suffolk Hospital NHS Foundation Trust, Bury St Edmunds IP33 2QZ, UK; 3 Division of Digestive Surgery, University Hospitals of Geneva, 1205 Geneva, Switzerland; 4 Medical School, University of Geneva, 1206 Geneva, Switzerland

**Keywords:** ulcerative colitis, surgery, proctectomy, surgical outcomes

## Abstract

**Background:** Reconstruction techniques after subtotal colectomy (STC) and end ileostomy for ulcerative colitis (UC), include ileal pouch-anal anastomosis (IPAA), ileorectal anastomosis (IRA) and continent ileostomy. **Aim:** To assess surgical strategies and outcomes after subtotal colectomy for UC by calculating the proportions of patients who had further surgery 10 years post-STC and those who did not undergo surgery but who were under surveillance, and histological analysis of pathology specimens from STC and proctectomy. **Methods:** Patients who had STC for UC from 2002 to 2018 were identified. Variables of interest were extracted from electronic records. Survival analysis on reconstruction surgery was performed using Kaplan–Meier curves. Curves were censored for loss from follow-up and death. Subtotal colectomy and proctectomy specimens were assessed by a pathologist for acute inflammation at the distal resection margin and within the resected bowel, and for dysplasia or cancer. **Results:** One hundred and ninety-two patients were included. Eighty-nine (46.3%) underwent proctectomy: eight had panproctocolectomy; thirty had completion proctectomy and the remaining fifty-one of the eighty-nine patients (27%) had IPAA. One patient who did not undergo a proctectomy had an ileorectal anastomosis. Sixty-one (69%) proctectomy specimens had active inflammation, with 29 (48%) including the resection margins. Of the 103 patients who did not have completion surgery, 72 (69%) were under surveillance as of August 2021. No patients in this non-operative group had developed cancer of the residual rectum at follow up. **Conclusions:** At 10 years after STC for UC, eighty-nine (46.4%) patients had proctectomy, of which fifty-two had IPAA (27%). However, no inflammation was found in the proctectomy specimen in one third of these patients. Therefore, it is possible that IRA may still have a role in the occasional patient with UC.

## 1. Introduction

Ulcerative colitis (UC) is defined as ‘chronic inflammatory disease characterised by mucosal inflammation starting distally in the rectum, with continuous extension proximally for a variable distance’ [1]. It is the most common inflammatory bowel disease (IBD) in the United Kingdom, with 12,369 new cases (out of 22,560 new cases of IBD) recorded from 2000 to 2018 [2,3]. Acute severe cases of UC requiring inpatient admission are defined by the modified Truelove and Witts criteria as severe diarrhoea (at least six episodes a day) with macroscopic blood in the stool, fever (temperature > 37.5 degrees), tachycardia (HR > 90), anaemia (Hb < 75) and ESR > 30 [4], although CRP is now used in current practice as it is not elevated in the elderly and is thus easier to define reducing CRP as disease remission [5]. The first-line management for patients having severe UC as per the modified Truelove and Witts criteria would be IV hydrocortisone 100 mg QDS or IV methylprednisolone 60 mg OD [4]. If disease remission is not achieved with IV steroids, then patients would next receive ciclosporin as ‘rescue therapy’ [4,6,7]. Infliximab is also an effective second-line treatment if remission is not achieved with IV steroids and has been shown to significantly reduce the need for colectomy but requires thorough screening of the past medical history as contraindications include active sepsis, congestive heart failure, latent tuberculosis and any demyelinating disease, such as multiple sclerosis [4,6,7]. When patients are still having more than eight bowel movements a day or three to eight bowel movements with CRP > 45 after three days of medical treatment, then they require a surgical review to determine whether they should be considered for emergency colectomy or for continuation of medical treatment [4]. The most common initial surgery for UC is currently subtotal colectomy (STC), which can either be performed in an elective or emergency setting [6,8]. Current indications for emergency STC for patients with UC include any clinical deterioration in patients’ symptoms and observations (e.g., stool frequency, body temperature); abdominal examination findings (e.g., perforation); blood results showing an increase in inflammatory markers (e.g., CRP); or any signs of toxicity that appear during intravenous immunosuppressive therapy, particularly Cushingoid features associated with therapy with excessive IV steroids [7]. The main indication for STC is the patient still having symptoms despite medical treatment or wishing to avoid long-term endoscopic surveillance to establish remission. However, other factors, such as whether the patient is on immunosuppressants, would also determine their fitness for surgery as excessive immunotherapy can prolong their post-op recovery and increase their risk of post-op complications [5]. To ensure maximal optimisation before and recovery post-surgery and therefore the improvement of long-term outcomes from colectomy, colorectal surgeons and gastroenterologists should work together to stabilise the patient and optimise their treatment [7].

Once a patient has undergone STC, the decision of whether or not to preserve the rectum depends on a variety of factors, including the degree of inflammation of the rectal stump (which correlates to patient’s future QoL), the presence of dysplasia or cancer, the degree of anal continence as well as the patient’s fitness for surgery and concerns about potentially reduced fertility [9]. Surgical treatment options currently include either a proctectomy with or without ileal pouch–anal anastomosis (IPAA), or the preservation of the rectum and creation of an ileorectal anastomosis (IRA). A proctectomy is only very rarely undertaken in an emergency setting due to the high risk of complications in an acutely unwell patient, and the timing of the proctectomy should be a joint decision between the patient and colorectal surgeon with MDT input [4]. Proctectomy with IPAA is currently preferred over IRA in the United Kingdom and is perceived as the gold-standard treatment for those wishing to have restoration of intestinal continuity since most patients requiring surgery have active inflammation in the rectal stump [10,11]. For instance, one study reported that 71 of 108 (65.7%) patients who had undergone a STC underwent proctectomy and IPAA due to the presence of active inflammation in the rectum [10]. It is popular as it means that patients do not require a permanent stoma, thus improving their quality of life [10]. However, IPAA is associated with a number of complications, such as the risk of pouchitis (50% in 10 years), small bowel obstruction (30% incidence in 10 years) and altered sexual function [10,11]. 

An alternative option in patients not willing to have a definitive stoma includes IRA. Data suggest that IRA can be suitable in selected groups of patients where the rectum has no active inflammation or dysplasia, and overall reduces post-operative morbidity [12,13]. IRA could also be a treatment option for women of reproductive age as it is less likely to cause sexual dysfunction or reduced fertility as it does not involve pelvic dissection [10]. However, IRA has not gained worldwide acceptance [11,14]. This is because many patients who would require IRA would often have residual inflammation of the rectal stump, which could threaten the anastomosis [14]. This means that patients who have had IRA would have to take continuous long-term topical 5-ASA, which would generally not be the case for patients with IPAA [14]. Ileorectal anastomosis is also not a definitive treatment option as it can lead to long-term complications, such as anastomotic stricture, poor function, altered QoL and neoplasia [9,11]. Patients therefore have to be followed-up over a longer period of time following IRA compared to IPAA [11]. It is therefore important to consider all the benefits and risks for both surgical options when deciding whether a patient is for surgery and which surgical strategy would be optimal in their case.

Our study had two main objectives:

1. To assess the long-term surgical outcomes of patients who had undergone a STC as treatment for UC through survival analysis from a tertiary centre;
2. Pathological analysis of the proctectomy specimens of patients who had proctectomy by means of analysing the degree of inflammation of the rectal stump, whether there was any inflammation present at the distal margins, and whether the specimens had any dysplasia to determine if IRA would have potentially constituted a suitable alternative surgical treatment strategy.

## 2. Methods

We performed a retrospective cohort study on patients of all ages who had undergone a TC at Addenbrooke’s Hospital, Cambridge University Hospitals NHS Foundation Trust, United Kingdom, from 31 December 2002 to 20 December 2018. Clinical evaluation study approval was provided by the Trust. Patients were identified based on a database of pathology specimens.

Inclusion and exclusion criteria were as follows:

- Inclusion: patients undergoing a STC for UC;
- Exclusion: patients who did not have an STC or patients who had a STC for a clinical indication other than UC.

Variables related to patient demographics (i.e., age, sex, BMI, weight and ASA classification), surgical indication, surgical procedure, and subsequent surgical history (metachronous proctectomy with or without IPAA or IRA) and oncological outcomes were extracted from electronic records. Survival analysis reporting on completion proctectomy and IPAA was performed using Kaplan–Meier curves. Curves were censored for loss from follow-up and death.

Continuous variables included patients’ age, weight and BMI, and the mean age is therefore presented as mean ± standard deviation. Categorical variables included patients’ sex, ASA classification, and indications for subtotal colectomy and proctectomy. Discrete data included the presence of inflammation in the subtotal colectomy specimens, proctectomy specimens, and whether any inflammation was present at the distal margin of the specimen. Data were extracted from a spreadsheet on Microsoft Excel 2010 listing all the pathological specimens and their analyses as well as from their electronic health records. Data were analysed on Microsoft Excel 2010, and STATA version 17 [12] was used to generate the Kaplan–Meier curves for survival analysis. We performed statistical analysis using Kaplan–Meier curves, and the remaining statistical measures are all descriptive. 

Pathological analysis of the operative specimens as documented on the aforementioned database of pathological specimens was carried out by multiple pathologists investigating the presence of active inflammation of the specimen, of dysplasia/cancer, and of any active inflammation at the distal resection margin for in both the STC and proctectomy specimens. 

## 3. Results

Our database originally identified seven hundred and four eligible patients for our study. After four hundred and ninety-seven patients were excluded as they did not meet our inclusion criteria of ‘patients who underwent STC for UC’, two-hundred and seven patients were identified. Fifteen further patients were then excluded due to missing data, leaving one hundred and ninety-two patients for inclusion. 

### 3.1. Demographics

One hundred and twenty one (58.5%) patients were male. The mean age at time of STC was 41.52 ± 17.60 years. The American Society of Anaesthesiologists (ASA) score [15] was II for ninety patients (46.9%), III for seventy-four patients (38.5%) and IV for twenty-eight patients (14.6%). No statistical significance was noted between these groups.

### 3.2. Long-Term Surgical Outcomes

**Figure 1:**
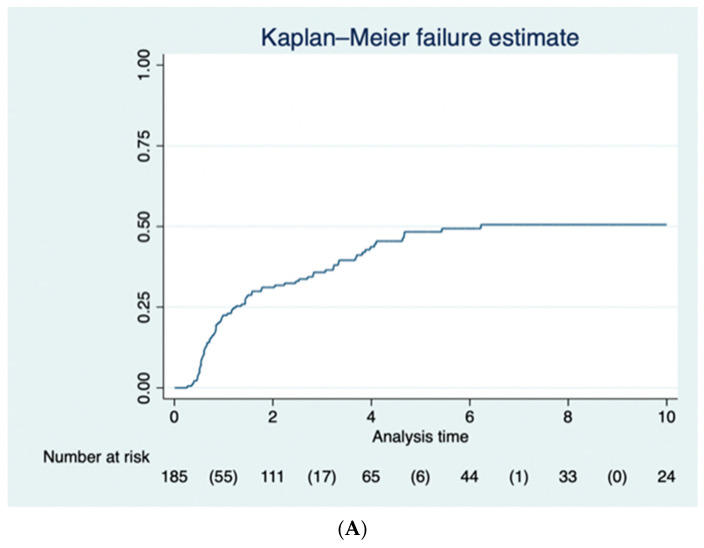

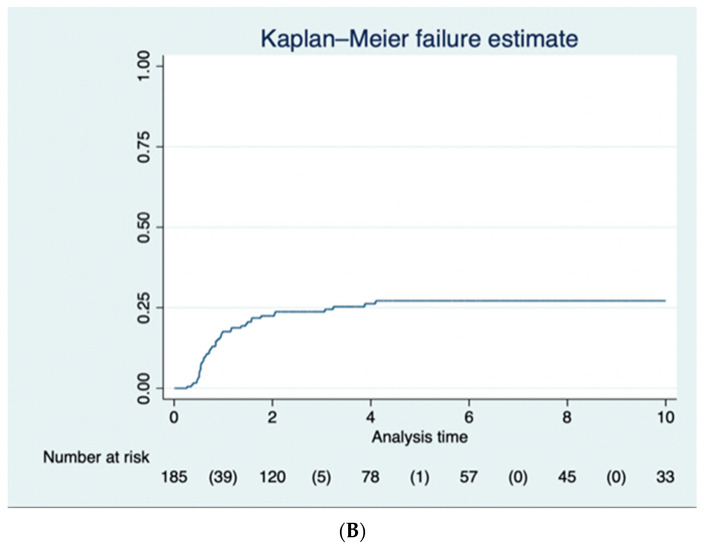
(**A**) The Kaplan–Meier curve for the 89 (46.3%) of patients who had a completion proctectomy following subtotal colectomy. (**B**) The Kaplan–Meier curve for the 52 (27.1%) of patients who benefitted from an ileoanal pouch following completion proctectomy.

Eighty-nine of the one hundred and ninety-two (46.3%) patients underwent proctectomy over the follow-up period: eight patients (4.2%) underwent panproctocolectomy, and eighty-one (42.1%) underwent proctectomy at a later date. The proportion of patients who had a proctectomy was 25.1% (95% CI 19.9–32.3%) at 1 year, 33.1% (95% CI 27.7–41.3%) at 2 years, 37.6% (95% CI 31.8–46.0%) at 3 years, 45.1% (95% CI 38.7–53.8%) at 4 years, 49.6% (95% CI 42.7–58.4%) at 5 years, and 51.8% (95% CI 44.7–60.8%) at 10 years after subtotal colectomy (Figure 1A). Reasons for proctectomy included persistence of symptoms after subtotal colectomy or wishing to avoid the need for long-term endoscopic surveillance in 70 patients (79%), acute exacerbation of ulcerative colitis in 16 patients (18%) and dysplasia in 3 patients (3%).

Fifty-two patients (60.5%) also had an IPAA over the follow-up period. The proportion of patients who had an IPAA was 20.4% (95% CI 15.3–27.0%) at 1 year, 25.2% (95% CI 19.5–32.1%) at 2 years, 26.5% (95% CI 20.7–33.6%) at 3 years, 28.9% (95% CI 22.7–36.3%) at 4 years, 29.9% (95% CI 23.5–37.5%) at 5 years, and 29.9% (95% CI 23.5–37.5%) at 10 years for patients with follow-up data available (Figure 1B).

One patient (0.52%) did not undergo proctectomy and instead had IRA.

Of the 103 patients (50.2%) who did not undergo any completion surgery and/or anastomosis, seventy-two (69% of them) were under surveillance at the end of follow-up period. None of the patients in this non-operative group had developed cancer of the residual rectum at follow up.

### 3.3. Evaluation of the Inflammation of the Residual Rectum

**Figure 2:**
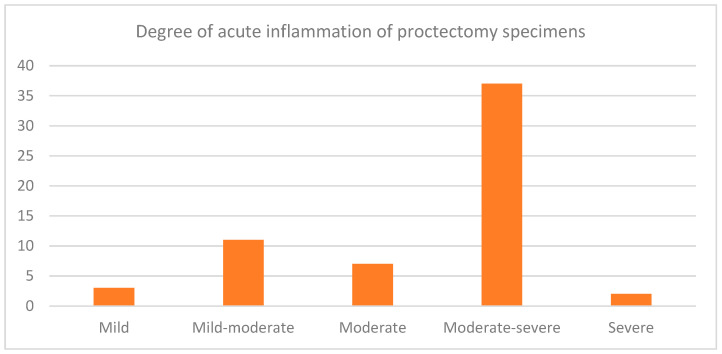
A bar graph demonstrating the proportions of proctectomy specimens with varying degrees of acute inflammation. Sixty of the eighty-nine proctectomy specimens (67.4%) showed active inflammation in the residual rectum, of which three were mild, eleven were mild to moderate, seven were moderate, thirty-seven were moderate to severe, and two were severe.

Eighty-nine patients underwent proctectomy over the follow-up period. The proportions of the proctectomy specimens showing degrees of active inflammation are shown in Figure 2. Forty-seven proctectomy specimens had chronic diversion colitis, two had dysplasia, and twenty-nine had active inflammation at the distal end of the resection margin. Overall, 52 of the 89 patients underwent IPAA, and 13 (25%) of these patients developed at least one recorded episode of pouchitis between 1 January 2006 and 30 March 2020.

## 4. Discussion

Ileorectal anastomosis is becoming an increasingly used restorative surgical treatment after STC for UC due to restoring continuity, reduced post-operative leakage, reduced post-operative morbidity and therefore a higher quality of life when compared to IPAA [13,14,16]. Of note, IRA has also been shown to have a reduced risk of sexual dysfunction and infertility as it does not involve pelvic dissection, therefore making it the more desirable option for women of reproductive age [11,13,14]. However, inflammation of the rectal stump would contraindicate IRA due to the increased risk of colorectal cancer [14], decreased quality of life in terms of ongoing symptoms of proctitis [6,17] and also increased risk of anastomotic leak [11]. Patients having IRA have also been shown to require a greater follow-up period post-operatively due to the increased failure rate and risk of complications as mentioned above [11,16]. 

In order to determine the proportion of patients who required completion proctectomy due to symptomatic residual disease in the rectal stump after STC, we analysed the surgical outcomes in a cohort of patients who had undergone STC at a tertiary centre over a period of 16 years. Similar studies have been carried out in Vermont, USA, and Amsterdam [18,19]. Moreover, in order to determine if IRA could have potentially constituted a viable alternative to IPAA, we analysed the post-operative histology specimens from these patients to assess the degree of inflammation in the rectal stump, including the presence of any active and/or chronic inflammation at the resection margins.

Our study demonstrated that eighty nine out of the one hundred and ninety two patients (46.3%) underwent a completion proctectomy, with 25.1% (95% CI 19.9–32.3%) at 1 year, 33.1% (95% CI 27.7–41.3%) at 2 years, 37.6% (95% CI 31.8–46.0%) at 3 years, 45.1% (95% CI 38.7–53.8%) at 4 years, 49.6% (95% CI 42.7–58.4%) at 5 years, and 51.8% (95% CI 44.7–60.8%) at 10 years after STC. A similar study from USA showed that 83 of 108 (76.9%) patients underwent a completion proctectomy within 18 months after their STC [18]. Seventy one (65.7%) of these patients underwent IPAA, whilst in our study, fifty two patients (60.5%) had IPAA over the follow-up period [18].

Our study also showed that sixty of the eighty-nine proctectomy specimens (67.4%) showed active inflammation in the residual rectum, of which three were mild, eleven were mild to moderate, seven were moderate, thirty-seven were moderate to severe, and two were severe. In total, 29 (32.6%) had active inflammation at the distal end of the resection margin. A similar study demonstrated that 167 (82%) patients had inflammation at the distal margin of the rectum [19]. However, in this study, nine patients (4.4%) had no active inflammation or diversion proctitis in the rectal stump, whilst our study showed that twenty-eight (33%) proctectomy specimens did not have any inflammation [19]. It is interesting to consider whether patients may therefore have been suitable for IRA in retrospect. Another study has also demonstrated how the rectal stump can be managed in patients with a preserved rectal stump after STC [20]. 

Ileorectal anastomosis has been demonstrated to be a safe and effective surgical treatment for patients with UC as long as they do not have any active inflammation in the rectum and are willing to take topical 5-ASA treatment long term [9,11,21]. However, IRA presents with complications, such as anastomotic leak, bowel obstruction and anastomotic strictures [14]. It also presents with high failure rates, with one study from a tertiary centre in Sweden reporting a failure rate of 34% mainly due to persisting proctitis, poor functioning of the anastomosis, or suspected or confirmed high-grade dysplasia or malignancy [22]. Another study reported that IRA has a higher failure rate than IPAA due to the increased likelihood of proctitis following IRA [11]. The studies cited in a recent systematic review exploring the use of IRA as a possible surgical treatment option for UC also gave the range of the failure rate between 18 and 27% [16]. This is reflected by more patients having to take anti-diarrhoeal medication following IRA compared to following IPAA [11]. However, patients having IRA were reported to have a better quality of life so perhaps IRA may continue to be a surgical treatment option for UC in the future [11]. 

We also identified 15 patients aged under 18 who had had a subtotal colectomy for UC between the 2014 and 2018, of which only 3 had a proctectomy. Data were only available for paediatric cases from 2014 to 2018, whilst the full dataset ranged from 2002to 2018. There is little research discussing surgical outcomes for UC in children, but the likely reasons include reduced morbidity and preserving fertility, as discussed above. One study explores ileoanal anastomosis as a possible treatment option for children who require the surgical management of UC as it was shown to have reduced post-operative morbidity and mortality and improved quality of life associated with a reduced likelihood of requiring a stoma [23].

There is very little research exploring the conversion of IRA to IPAA due to IRA failure, but one study investigating eight patients has shown that the main indications for conversion to IPAA are the same as the reasons why IRA fail, including intractable proctitis (five patients), colorectal cancer (two patients) and rectal dysplasia (one patient) [19]. This highlights the need for careful patient selection and shared decision making when considering restorative surgery using IRA or IPAA [6,24]. 

Of the 103 patients who did not undergo completion surgery, 72 (69.2%) were under surveillance as of August 2021. The age range of the patients who were under surveillance was 10–83 years old, and the age range of the patients who were not under surveillance was 18–76 years old; there was no statistical significance between the two age ranges. However, all fifteen of the paediatric cases were in a surveillance programme. Many patients were lost to follow-up and reasons include relocating to different parts of the country/world and patients not attending their follow-up appointments. 

Reassuringly, none of the patients in this non-operative group had developed cancer of the residual rectum at follow up. 

There have been various studies exploring the efficacy of an endoscopic surveillance programme post-STC in reducing the risk of rectal cancer, with one cohort study from Ontario, Canada, demonstrating that endoscopic surveillance post-STC reduces the risk of developing rectal cancer, with a hazard ratio of 0.3–0.4 for patients with regular endoscopic surveillance [3]. However, another study highlights the risks of using endoscopic surveillance as a method of reducing cancer risk, stating that regular endoscopic surveillance adds a huge cost to health services, not to mention over diagnosing cases of colorectal cancer and putting patients through unnecessary treatment [25,26]. A risk-benefit approach would have to be undertaken to determine the efficacy of endoscopic surveillance, and the frequency of endoscopic surveillance would play a role in this. 

With different UC patients having different risk profiles for colorectal cancer, the frequency of surveillance would have to vary depending on the risk profile. A systematic review exploring endoscopic surveillance highlighted that all patients should be under surveillance with endoscopy and biopsy, but the different studies in the review reported different intervals of follow up: two studies (20%) recommended an interval of 6 months to a year, five recommended yearly surveillance (50%), one (10%) recommended surveillance every 2 years, and the remaining three (30%) recommended that the frequency of surveillance should be based on patient risk stratification [27,28]. Another study highlighted that the high-risk patients with a family history of malignancy had yearly endoscopic surveillance, whilst medium-risk patients had endoscopic surveillance every 2–3 years, and low-risk patients had endoscopic surveillance every 5 years [25].

Ironically, adherence to the surveillance programme was lower in the high-risk patients and higher in the lower-risk patients [29,30]. This may have been due to the frequency and costs associated with travelling for appointments as well as the discomfort associated with bowel preparation for endoscopic surveillance [29,30]. 

The main strength of this study is the long-term follow-up of patients after STC and review of the degree of inflammation in the proctectomy specimen. Having a long-term follow-up period allowed us to determine the proportion of patients who underwent further operation by means of proctectomy, IPAA or IRA, and the proportion of patients who did not undergo any further operation but who were under surveillance.

Post-operative analysis of the proctectomy specimens also enabled us to determine any indication for further surgery and whether the patients may have been suitable for IRA in retrospect.

However, our study did pose the following limitations:

(1) A modest sample size caused by the monocentric design of the study and the low incidence of UC requiring STC. With the hospital’s electronic record only developing in 2014, it also made it difficult to obtain data before this period. The pathology data was also on a different system to the main hospital record, which limited the data that we could obtain from the database of histopathology specimens. This also made it difficult to interpret whether patients before 2014 had adhered to follow-up with endoscopic surveillance.
(2) A follow-up period limited to a maximum of 16 years. Perhaps if we had included earlier, we could have used a longer follow-up period. However, the incidence of metachronous surgery after STC plateaued quite early in our study, and thus, extending the follow-up period may not have necessarily added further useful data.
(3) Our study was designed as a retrospective cohort study, resulting in a possible increased loss to follow-up if patients sought treatment in another centre. A prospective cohort study may have enabled us to keep track of patient adherence to follow-up.
(4) Limited access to data made it very difficult to perform a full analysis on the risk factors for proctectomy. This was particularly the case with any data before the installation of the current electronic health record in 2014.

Suggestions for future studies could include a multicentre study to determine whether this shows similar findings, continuing to follow-up these patients prospectively, and repeating the study in the future to determine whether there is a change in the proportions of patients that opt for completion surgery vs. endoscopic surveillance and lifelong medical treatment whilst technology, such as robotic surgery, is developing in the setting of pelvic surgery and IBD [31].

## 5. Conclusions

Our study highlights that IRA could possibly constitute a safe alternative to proctectomy and IPAA in selected patients willing to have restorative surgery after STC for UC, given that one third of the patients in our study did not have inflammation in the rectum as per the pathological specimens. However, IRA can present with novel complications that IPAA may not present, both immediately and in the long term, including a higher risk of ongoing proctitis and subsequent malignancy. Being under surveillance and attending follow-up can reduce the risk of these complications arising. 

## Data Availability

Data were obtained from the patient medical records at Cambridge University Hospitals NHS Foundation Trust, Cambridge, UK.
